# 1510. Evaluation of Quantitative (1,3)-β-D-glucan in Prediction of *Pneumocystis jirovecii* Pneumonia in Patients Living with HIV in New York

**DOI:** 10.1093/ofid/ofad500.1345

**Published:** 2023-11-27

**Authors:** Philip Yune, Uriel Felson, Melissa Fazzari, Erika Orner, Robert Grossberg

**Affiliations:** North Shore University Hospital, Northwell Health, Manhasset, New York; Montefiore Medical Center, Bronx, New York; Albert Einstein College of Medicine, Bronx, New York; Montefiore Medical Center, Bronx, New York; Albert Einstein College of Medicine/Montefiore Medical Center, Bronx, New York

## Abstract

**Background:**

(1,3)-β-D-glucan (BDG) has been utilized as an indirect marker for *Pneumocystis jirovecii* pneumonia (PJP). The clinical utility of BDG, however, is limited because of uncertainty in interpreting quantitative values. A correlation was drawn between quantitative BDG and the diagnosis of PJP in hospitalized patients living with human immunodeficiency virus (HIV) at four affiliate campuses of the Montefiore Health System in the Bronx, NY.

**Methods:**

All *Pneumocystis* direct fluorescent assays (DFA), polymerase chain reactions (PCR) either from bronchoalveolar lavage (BAL) fluid or sputum, and quantitative BDG assays either from serum or BAL fluid were retrieved from the Montefiore microbiology laboratory (date range: 8/1/18 - 8/31/22). Results from hospitalized patients with HIV whose CD4 count within 3 months were available, with either PJP DFA or PCR result within 7 days of serum BDG and lactate dehydrogenase (LDH) test were selected (n = 146). CD4 count was grouped into < 50 (n = 106), 50-100 (n = 15), 100-200 (n = 16), and >200 (n = 9). BDG, LDH, and CD4 were considered as input variables in a logistic regression model for true PJP status. The area under the curve (AUC) of the empirical receiver operating characteristic (ROC) curve with 95% confidence intervals (CI) was estimated based on trapezoidal area. Comparisons between ROC curve areas were performed via contrast matrix to take differences of the AUC of the empirical ROC curves and chi-square testing.

**Results:**

Of the 146, 29 had PJP detected and 117 did not. When BDG only was considered, the AUC of the ROC curve was 0.78. When LDH quartiles (cutoffs: 232, 334, and 480 U/L) were considered with BDG, there was a statistically significant increase in AUC (0.84 vs. 0.78; *p* = 0.025). Adding CD4 group did not influence AUC.

Figure 1

**Figure:**
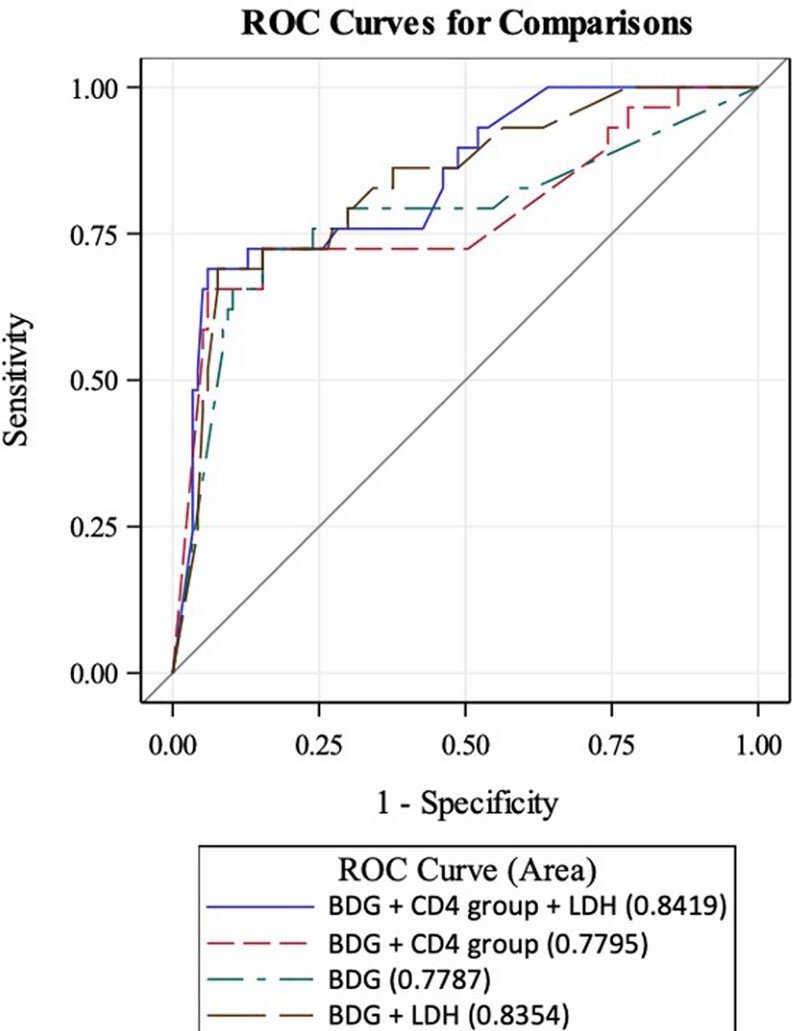

ROC curves for BDG only, BDG with CD4 count, BDG with LDH quartile, and BDG with CD4 group and LDH quartile combined

Table 1

**Figure:**
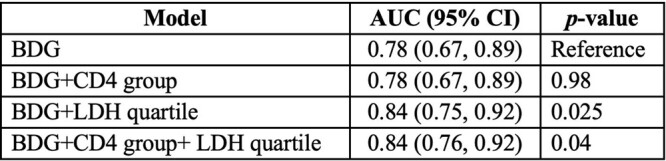

Area under the curve calculated for the ROC curves for BDG only, BDG with CD4 count, BDG with LDH quartile, and BDG with CD4 group and LDH quartile combined

**Conclusion:**

The test characteristics (AUC) improved when LDH quartiles were used in conjunction with BDG, suggesting a better prediction of PJP diagnosis when BDG and LDH were considered together. This result supports further investigation of clinical markers of PJP given the limitations of establishing a microbiologic diagnosis. Further steps of the investigation will involve analyzing if other clinical factors including HIV viral load, oxygen saturation, and radiographical reading, influence predictability of PJP.

**Disclosures:**

**All Authors**: No reported disclosures

